# Comparative proteomic investigation of drought responses in foxtail millet

**DOI:** 10.1186/s12870-018-1533-9

**Published:** 2018-11-29

**Authors:** Jiaowen Pan, Zhen Li, Qingguo Wang, Anna K. Garrell, Min Liu, Yanan Guan, Wenqing Zhou, Wei Liu

**Affiliations:** 1 Biotechnology Research Center, Shandong Academy of Agricultural Sciences; Key Laboratory of Genetic Improvement, Ecology and Physiology of Crops, Jinan, 250100 Shandong China; 2 Crop Research Institute, Shandong Academy of Agricultural Sciences, Jinan, 250100 Shandong China; 3 College of Life Sciences, Shandong Normal University, Jinan, 250014 Shandong China; 4 Shandong Agriculture and Engineering University, Jinan, 250100 Shandong China; 5 Boragen Inc, Durham, North Carolina 27709 USA

**Keywords:** Foxtail millet (*Setaria italica* L.), Drought stress, Comparative proteomics, Expression pattern, Western blot, qRT-PCR

## Abstract

**Background:**

Foxtail millet (*Setaria italica* L. *P. Beauv*) has been considered as a tractable model crop in recent years due to its short growing cycle, lower amount of repetitive DNA, inbreeding nature, small diploid genome, and outstanding abiotic stress-tolerance characteristics. With modern agriculture facing various adversities, it’s urgent to dissect the mechanisms of how foxtail millet responds and adapts to drought and stress on the proteomic-level.

**Results:**

In this research, a total of 2474 differentially expressed proteins were identified by quantitative proteomic analysis after subjecting foxtail millet seedlings to drought conditions. 321 of these 2474 proteins exhibited significant expression changes, including 252 up-regulated proteins and 69 down-regulated proteins. The resulting proteins could then be divided into different categories, such as stress and defense responses, photosynthesis, carbon metabolism, ROS scavenging, protein synthesis, etc., according to Gene Ontology annotation. Proteins implicated in fatty acid and amino acid metabolism, polyamine biosynthesis, hormone metabolism, and cell wall modifications were also identified. These obtained differential proteins and their possible biological functions under drought stress all suggested that various physiological and metabolic processes might function cooperatively to configure a new dynamic homeostasis in organisms. The expression patterns of five drought-responsive proteins were further validated using western blot analysis. The qRT-PCR was also carried out to analyze the transcription levels of 21 differentially expressed proteins. The results showed large inconsistency in the variation between proteins and the corresponding mRNAs, which showed once again that post-transcriptional modification performs crucial roles in regulating gene expression.

**Conclusion:**

The results offered a valuable inventory of proteins that may be involved in drought response and adaption, and provided a regulatory network of different metabolic pathways under stress stimulation. This study will illuminate the stress tolerance mechanisms of foxtail millet, and shed some light on crop germplasm breeding and innovation.

**Electronic supplementary material:**

The online version of this article (10.1186/s12870-018-1533-9) contains supplementary material, which is available to authorized users.

## Background

Foxtail millet (*Setaria italica* L.) is an ancient crop in the subfamily of *Panicoideae*, and is distributed worldwide in arid and semi-arid regions. It originated in North China, and was domesticated more than 8,700 years ago. As foxtail millet grains are rich in protein and minerals, especially when compared to those of rice, wheat, and maize, it was named first among the “Five Grains of China” due to its high nutritional values [1].

Foxtail millet possess most noticeable morphological and anatomical attributes, such as dense root distribution, thick cell wall, small leaf area and epidermal cell arrangement, which endow it with strong drought tolerance and high water use efficiency, and further allow it to be primarily cultivated in arid, semi-arid, and barren regions. Today, foxtail millet is attracting more attention in agricultural production, especially as global warming and lacking water resources become increasingly severe in the world [2].

Additionally, foxtail millet carries attractive qualities such as a small diploid genome (~490 Mb), inbreeding nature, less repetitive DNA, short growing cycle and abiotic stress-tolerance [3]. As opposed to other proximal plants, such as pearl millet, switchgrass, and napiergrass, these features highlight it as a model crop for exploring the mechanisms of drought tolerance, evolutionary genomics, architectural traits, C4 photosynthesis and the physiology of bioenergy crops [4]. Recently, the genome sequences of foxtail millet cultivars“Yugu1” and “Zhanggu” have been sequenced and submitted by the US Department of Energy Joint Genomic Institute and Beijing Genomics Institute (BGI) of China, respectively [3, 5].

After the genomic sequence of foxtail millet was released, the vital stress-related gene families, such as the *SiNAC*, *SiWD40* and *SiALDH* gene families, were systematically analyzed and identified [6–8]. The *SiPLDa1*, *SiDREB2*, *SiNAC*, *SiOPR1*, and *SiAGO1b* genes were all reported to mediate various stress responses and developmental processes during dehydration stress [6, 9–12]. Deep sequencing technology was also used to investigate the genome-wide transcriptome reconfiguration of foxtail millet under drought stress, and a great number of differentially expressed genes (2,824), long noncoding RNAs (lncRNAs) and small interfering RNAs (siRNAs) were identified [13]. Under dehydration stress, 105 and 84 differentially expressed genes were identified in foxtail millet roots and shoots, respectively, and the responses of genes involved in gluconeogenesis and glycolysis pathways took place earlier in roots compared to shoots. Furthermore, the protein degradation pathway may also perform a key role in drought tolerance of foxtail millet [11].

Although drought-responsive genes and noncoding RNAs (ncRNAs) were identified, there have been hardly any systematic investigation summaries of protein profiling for drought stressed foxtail millet. Protein profiling will contribute to the systematic scrutiny of changes in protein levels and activities, and provide information about which proteins may participate in certain biological processes. Recently, tandem mass tags (TMT), combined with liquid chromatography−quadruple mass spectrometry (LC−MS/MS) analysis, has been utilized as an useful quantitative proteomic technique, which facilitates simultaneous identification and relative quantification of proteins with great efficiency and accuracy. This method is also widely used for quantitative comparative analysis of plant proteomes [14]. In this study, the TMT combined with LC-MS/MS-based proteomic approach was used, and the differentially expressed proteins in foxtail millet seedlings after drought treatment were quantitatively identified. There were 2474 differential proteins that were quantitatively identified, among which, 321 drought responsive proteins were identified. Bioinformatic analysis revealed that these differential proteins may take part in various biological processes. These biological processes may function synergistically by initiating different response mechanisms on the protein level to reconfigure and achieve new homeostasis in drought conditions. Our results begin filling the gap in our knowledge regarding the proteomic activity and regulated response mechanisms under drought conditions in foxtail millet, which will further deepen the understanding of the physiological and molecular basis of stress tolerance in crops.

## Materials and methods

### Plant materials and growth conditions

The foxtail millet variety, Yugu1, which is known to be a drought resistant variety and whose genome has been sequenced, was used for all experiments [15]. Plastic pots (21 cm in diameter × 21 cm in height) were used as experimental units. Each pot was filled with 3-kg soil consisting of a mixture of nutrient soil and loamy sand in a ratio of 1:1. Plants were grown in greenhouse with well-watered conditions under 30/25 °C day/night cycle with a 14-h photoperiod for three weeks. The drought treatments were performed as previously described [16]. Soil moisture of well-watered and drought-treated experimental units was controlled at 60–70% and 20-30% of field capacity respectively, and the treatments lasted for 7 days. The pots were randomized in four replicates between the two treatments. After drought treatments, seedlings were immediately harvested and frozen in liquid nitrogen and stored at −80 °C for protein and RNA extraction, and then to perform proteomic, western blot and gene expression analysis.

### Protein extraction and trypsin digestion

Protein extraction and trypsin digestion were performed according to a previously established method [17] with slight modification. Samples were first grinded in liquid nitrogen, then the tissue powder in lysis buffer was ultra-sonicated three times. The remaining debris was removed after centrifugation, and the protein was precipitated with 15% TCA and washed with cold acetone. The precipitates were re-suspended in buffer made of 100 mM TEAB and 8 M urea at pH 8.0. The protein concentration was determined using 2-D Quant kit (GE Healthcare B80648356 Piscataway USA).

For protein digestion, the protein solution was reduced with 10 mM DTT and alkylated with 20 mM Iodoacetic acid. After that, the protein samples were diluted by adding 100 mM TEAB with urea concentration less than 2M. Finally, the protein was digested at 1:50 and 1:100 trypsin-to-protein mass ratios with two replicates of each. For each sample, approximately 100 μg of protein were digested for the following experiments.

### TMT labeling and HPLC fractionation

After trypsin digestion, the peptide was desalted by Strata X C18 SPE column (Phenomenex) and vacuum-dried. The peptide was reconstituted using 6-plex TMT kit according to the manufacturer’s protocol and the sample was then fractionated according to protocol of [17].

### LC-MS/MS analysis

The peptides were dissolved in 0.1% formic acid (FA), and then directly loaded onto a reversed-phase pre-column (Acclaim PepMap 100, Thermo Scientific). The peptide separation was performed using a reversed-phase analytical column (Acclaim PepMap RSLC, Thermo Scientific). LC-MS/MS analysis was performed according to protocol of [17].

### Database searching

The resulting MS/MS data were processed using Mascot search engine (v.2.3.0). Tandem mass spectra were blasted against *Uniprot_foxtail_4555* (http://www.uniprot.org/taxonomy/4555) database concatenated with reverse decoy database. Trypsin/P was specified as the cleavage enzyme allowing up to two missing cleavages. Mass error was set to 10 ppm for precursor ions and 0.02 Da for fragment ions. Carbamidomethyl on Cys, TMT6plex (N-term) and TMT6plex (K) were specified as the fixed modifications, and oxidation on Met was specified as the variable modification. FDR was adjusted to <1% and peptide ion score was set to >20.

Gene Ontology (GO) annotation proteome was derived from the UniProt-GOA database (www. http://www.ebi.ac.uk/GOA/). The proteins were classified by Gene Ontology annotation based on three categories: biological process, cellular component and molecular function. The functional description of identified protein domains was annotated by InterProScan (a sequence analysis application) based on protein sequence alignment method, using the InterPro domain database. Kyoto Encyclopedia of Genes and Genomes (KEGG) database (http://www.genome.ad.jp/kegg/) was used to annotate protein pathway: first, by using the KEGG online service tool, KAAS, to annotate e the protein’s KEGG database description, then by mapping the annotation results on the KEGG pathway database using KEGG online service tool, KEGG mapper. Wolf-psort (http://www.genscript.com/wolf-psort.html) was used to predict the protein’s subcellular localization. The Protein-Protein Interaction analysis was performed according to [18].

### Physiological parameters measurements

The measurements of physiological parameters, such as antioxidant enzyme activity and proline and soluble sugar content, were performed as described previously [19]. The Glycine betaine (GB) content was measured according to [20]. The detections of spermine and spermidine were carried out according to the method of [21].

### Western blot analysis

Total proteins were extracted from foxtail millet seedlings in extraction buffer containing 100 mM Tris pH 8.0, 5 mM EDTA, 1 mM PMSF and 0.2% β-mercaptoethanol. Protein extracts were separated on 12% SDS−PAGE gels and then transferred to a PVDF membrane using a Mini Trans-Blot cell (Beijin Jun Yi electrophoresis equipment, China). The membranes were first blocked with 10% bovine serum albumin in TBST buffer (100mM NaCl_2_, 20mM Tris pH 8.0 and 0.5% Tween-20) for 2h, and then incubated with polyclonal antibodies at a 1:1000 dilution for another 2h at room temperature. After washing three times with TBST buffer, the membranes were incubated with secondary antibody of horseradish peroxidase (HRP)-conjugated goat anti-rabbit IgG at a 1:1000 dilution for 2h. After three times further washes in TBST buffer,the membranes were visualized with a 3,3′-diaminobenzidine (DAB) detection system (Sangon Biotech, China). The western blot analysis was repeated twice. The SiActin (XM_004978702) was used as the internal control to quantify protein loading of different samples. Antibody production was conducted as described above. The peptides of SiPPR (XM_004978236) (residues 186-508) and SiRLK (XP_004956304.1) (residues 1-386) were expressed and purified as described [22]. The purified peptide was injected into rabbits for polyclonal antibody preparation. Using these polyclonal antibodies, only one band was detected at the corresponding position in the Western Blot. The antibodies for SiCAT (XM_004985783), SiHSP70 (XM_004981194), SiTuBulin (XM_004981865) and Actin were purchased from Agrisera, Sweden(product numbers were AS09 501, AS08 371, AS10 680, and AS13 2640, respectively.

### RNA isolation and quantitative real-time PCR

Total RNA from foxtail millet was extracted with Trizol reagent (TaKaRa) according to the manufacturer’s instructions. The RNA electropherogram is shown in Additional file 1: Figure S1. First strand cDNAs were synthesized using the First Strand cDNA Synthesis kit (TaKaRa). Quantitative Real-time PCR was performed in 7500 real-time PCR machine (Applied Biosystems) using the FastStart Universal SYBR Green (Roche) Master. The FastStart Universal SYBR Green (Roche) Master is supplemented with ROX reference dye for background noise correction. Each PCR reaction was carried out with gene-specific primers in a total volume of 20μL containing 10μL SYBR Green Master mix, 0.5μM gene-specific primers, and appropriately diluted cDNA. The foxtail millet actin gene *SiActin* was used as the internal reference [7]. All primers were annealed at 56 °C. Each PCR reaction was repeated three times independently. Relative gene expression was calculated according to the delta-delta Ct method [7]. All primers are listed in Additional file 2: Table S1.

## Results

### Identification and quantification of drought-responsive proteins of foxtail millet

**Fig. 1:**
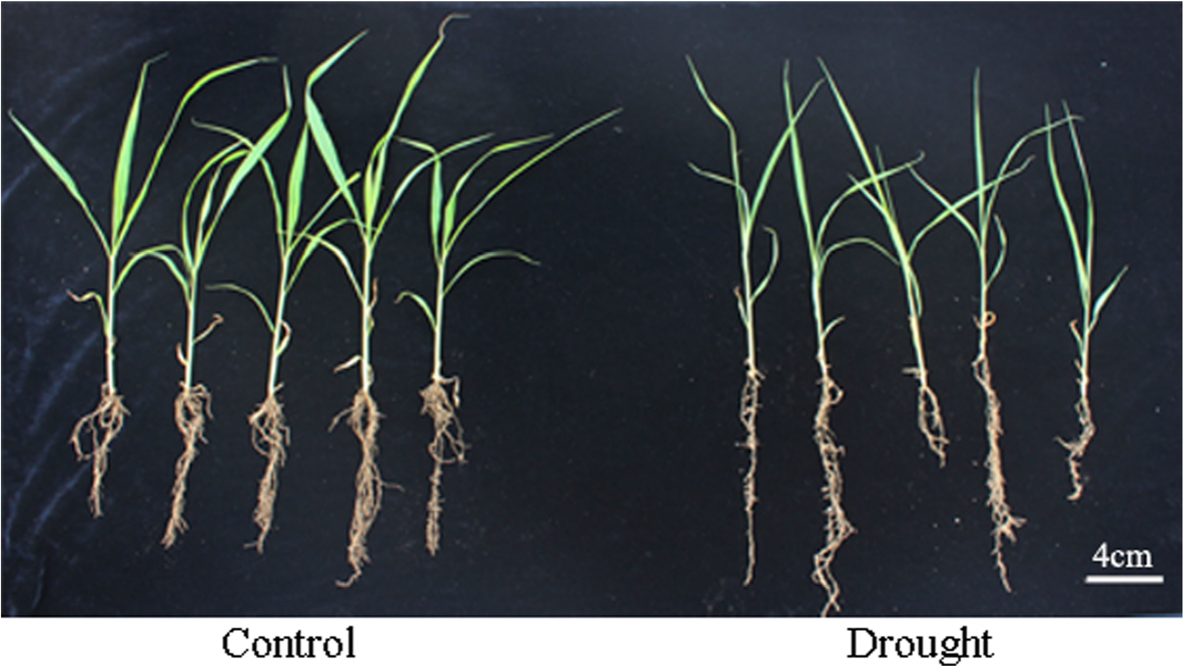
The phenotype of foxtail millet seedlings under drought stress

**Fig. 2:**
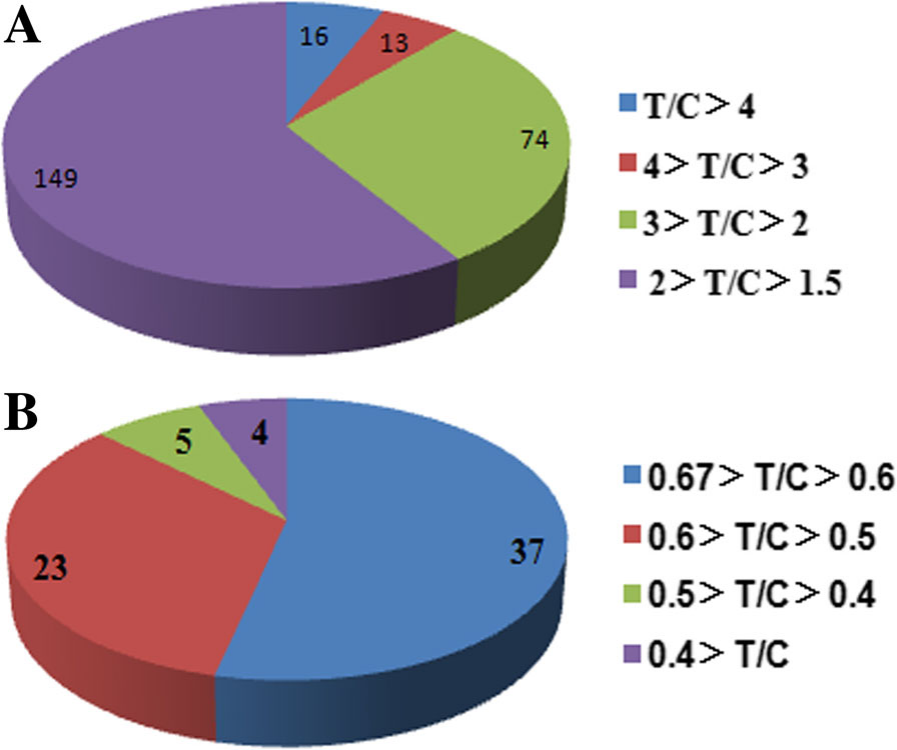
Pie chart showing the distribution of differentially expressed proteins. T/C Ratio: relative fold change abundance of proteins in Foxtail millet under drought stress compared with control (cutoff of over 1.5 for increased expression and less than 1/1.5 (0.67) for decreased expression). **a**: Up-regulated proteins, **b**: Down-regulated proteins

After natural drought treatment for 7 days, the foxtail millet seedlings showed stunted growth, and yellowish, wilting and curled leaves compared to those of the untreated control (Fig. 1). These seedlings were then used for quantitative proteomic analysis. A total of 4074 annotated proteins were identified in two biological replicates (Additional file 3: Table S2). Among the identified 4074 proteins, 2474 proteins were found in all four replicates and could be quantified (Additional file 4: Table S3). In published reports, the cutoff values of 1.2- to 1.5-fold change threshold and p-value (p < 0.05) was adopted [17, 23–25]. We adopted more stringently threshold of fold changes (cutoff of over 1.5 for increased expression and less than 1/1.5 (0.67) for decreased expression) and p-value<0.01 to assess significant changes according to previous report [26, 27]. With a threshold of fold changes (cutoff of over 1.5 for increased expression and less than 1/1.5 (0.67) for decreased expression) and p-value <0.01, 321 proteins with significant abundance variations were obtained, of which the abundance of 252 proteins increased while the abundance of the other 69 proteins decreased (Fig. 2, Additional file 5: Table S4). In the proteins with increased expression, 16 proteins showed increased levels of over 4 folds compared to that of the control, 87 showed increased levels between 2 and 4 folds than that of the control, and the remaining 149 proteins showed an increase of less than 2 folds. The variations of 60 decreased proteins ranged from 0.67-0.5 fold compared to that of control, while only 9 proteins among these changed by less than 0.5 fold (Fig. 2).

### Overview of quantitative proteomics analysis

**Fig. 3:**
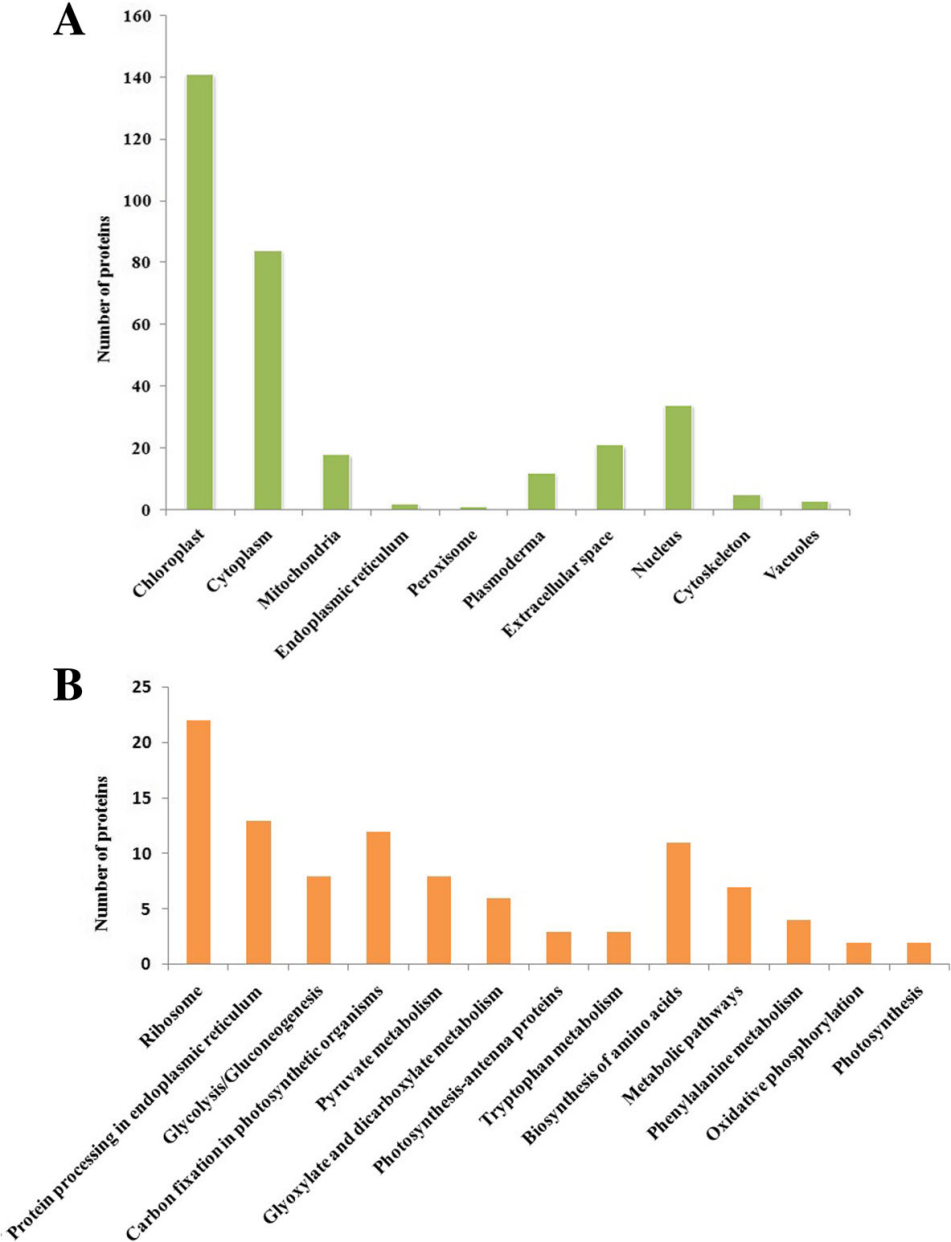
Categories of differentially expressed proteins. **a**: The subcellular localization, **b**: The KEGG Pathway Annotation

Referring to the classification criteria of the categories, these drought-responsive proteins could be further classified into different sub-categories. The subcellular localization analysis showed that these proteins were mainly localized in the chloroplast, cytoplasm, mitochondria, endoplasmic reticulum (ER), peroxisome, plasmodesma, extracellular space, nucleus, cytoskeleton, and vacuoles (Fig. 3a). Furthermore, about 140 proteins that accounted for 43.6% of the 32l differential proteins were mainly localized in the chloroplast. The results indicated relatively comprehensive distributions and functions of these identified proteins, and displayed the importance of chloroplast-related proteins and biological processes under drought response processes. To further investigate the pathways the identified proteins are involved in, KEGG analysis was also performed. Results suggested that these proteins were mainly enriched in ribosome-related protein processing in the endoplasmic reticulum, carbon fixation, glycolysis/gluconeogenesis, pyruvate metabolism and amino acid biosynthesis (Fig. 3b).

### Network of protein-protein interaction

**Fig. 4:**
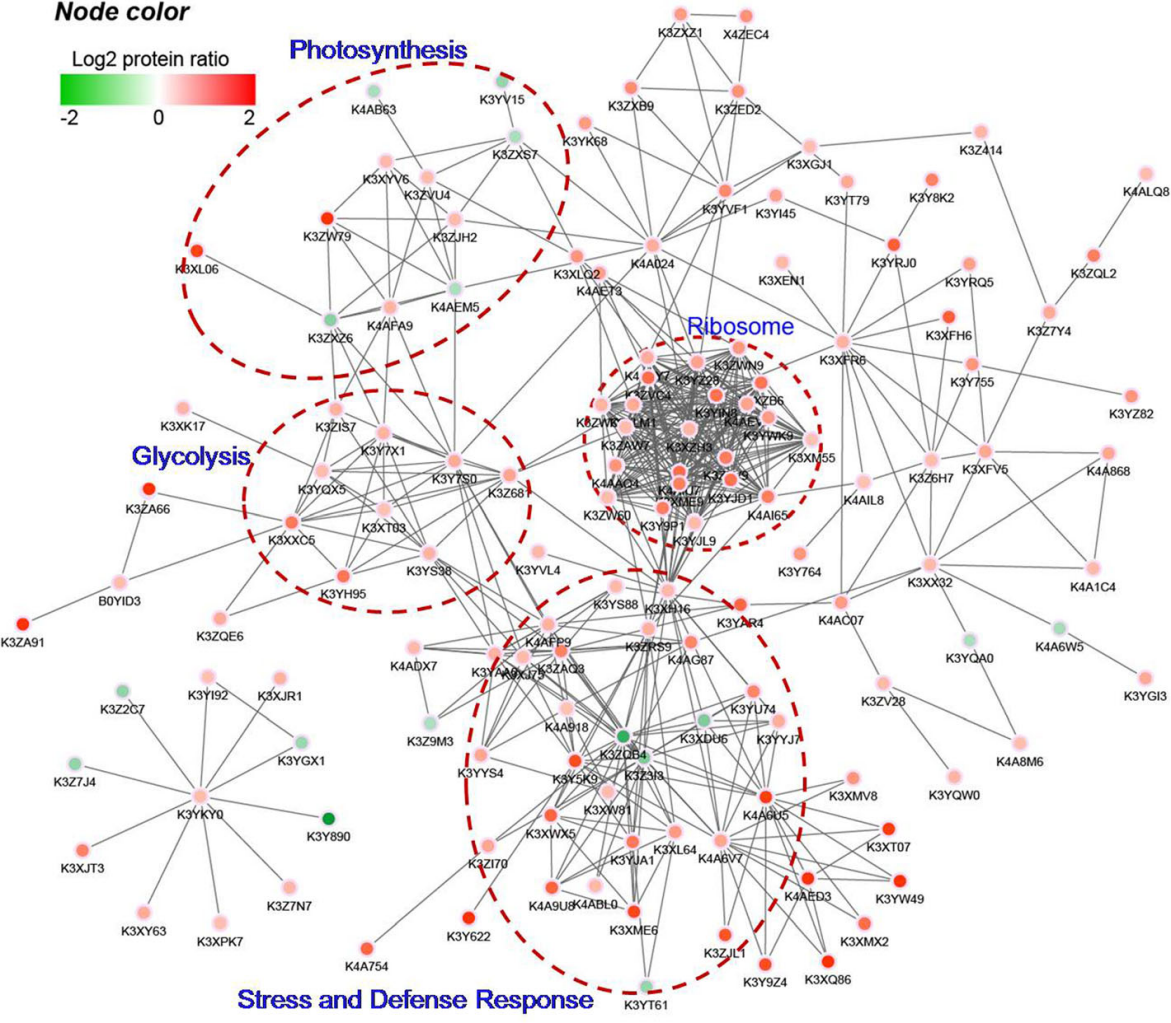
Analysis for Protein-protein interaction network for the different expressed proteins

To better understand how these diverse pathways interrelate in foxtail millet under drought conditions, the protein-protein interaction network was assembled using the STRING database and Cytoscape software. As shown in the interactome figure, there are 137 nodes and 317 interactions with a combined score higher than 0.70 (Fig. 4, Additional file 6: Table S5). Four concentrated clusters, which include most of the drought responsive and interactive proteins, were highlighted by the dotted circles (Fig. 4). The functions of the proteins in these four clusters are generally focused on stress and defense response, photosynthesis, glycolysis, and protein synthesis; the abundance of most of these proteins increased, which displays the pivotal response of these proteins under drought conditions. Intensive study of the variations and correlations of these crucial nodes and their encompassed proteins in foxtail millet are undergoing.

### Functional classification of differential proteins

The identified drought-responsive proteins were further classified into seven major categories according to the functional categories [28, 29]: stress and defense response, photosynthesis, carbon metabolism, ATP synthesis, protein biosynthesis, folding and degradation, metabolism-related proteins, and cell organization-related proteins (Table 1).

**Table 1:** Differentially Expressed Proteins in Foxtail millet under drought stress

Protein accession	Protein name	T/C Ratio
A Stress and defense response		
K3Z9P0	late embryogenesis abundant (LEA) protein, group 3-like	3.7
K3XLP0	late embryogenesis abundant (LEA) protein, group 3-like	2.3
K3XYM7	late embryogenesis abundant (LEA) protein D-34-like	2.3
K3YJU5	late embryogenesis abundant (LEA) protein D-34-like	1.8
K3ZK82	abscisic stress-ripening protein (ASR) 2-like	2.8
K3YB35	abscisic stress-ripening protein (ASR)	2.0
K3YAY4	Non-specific lipid-transfer protein (nsTLP)	8.5
K3ZKC1	Non-specific lipid-transfer protein (nsTLP)	4.4
K3XN75	Non-specific lipid-transfer protein (nsTLP) 2B-like	2.8
K3ZKB1	Non-specific lipid-transfer protein (nsTLP)	2.4
K3YJA1	14-3-3-like protein GF14-C-like	2.4
K3XL64	14-3-3-like protein GF14-C-like	1.9
P19860	Bowman-Birk type major trypsin inhibitor	4.0
K4AJ19	Bowman-Birk type trypsin inhibitor-like	2.5
K3YAC0	Bowman-Birk type bran trypsin inhibitor-like	1.7
K3ZLV9	cysteine proteinase inhibtor 8-like	2.8
K3ZP55	cysteine proteinase inhibitor 8-like	2.0
K3ZRS9	serine/threonine-protein phosphatase 2A	1.7
K3XH16	DEAD-box ATP-dependent RNA helicase 20-like	1.6
K3YXA4	defensin-like protein 2-like	4.1
K4AG87	Superoxide dismutase (SOD) [Cu-Zn]	2.2
K3YYS4	peroxiredoxin-2E-2, chloroplastic-like	1.8
K3XJT3	peroxidase (POD) 72-like	2.3
K3XY63	peroxidase (POD) 3-like	1.7
K3XJR1	peroxidase (POD) 72-like	1.6
K3Z7N7	peroxidase (POD) 2-like	1.6
K3YTW2	peroxidase (POD) 2-like	1.6
K3XPK7	peroxidase (POD) 24-like	1.5
K3XW81	Catalase (CAT)	1.5
K4A918	Catalase (CAT)	1.5
K4AE60	L-ascorbate peroxidase (APX)1, cytosolic-like	1.9
K4AET3	glutathione S-transferase (GST) F8, chloroplastic-like	1.8
K3Z9K9	glutathione S-transferase (GST) DHAR2-like	1.6
K3YAR4	glutaredoxin-C6-like	2.7
K3ZAQ3	thioredoxin	2.3
K3YAA0	thioredoxin M2, chloroplastic-like	1.6
K4AFP9	thioredoxin F, chloroplastic-like	1.6
K3Z7G0	aldo-keto reductase	1.6
K4AAI8	GDP-D-mannose 3,5-epimerase (MEG)	1.8
K3XEC7	respiratory burst oxidase homolog protein B-like	1.6
K3ZQB4	leucine-rich repeat receptor-like serine/threonine-protein kinase BAM1-like	0.3
K3Z3I3	leucine-rich repeat receptor-like protein kinase	0.5
K3YT61	receptor-like protein kinase	0.5
K3ZRC5	G-type lectin S-receptor-like serine/threonine-protein kinase	0.5
K3Y7I7	SNF1-related protein kinase regulatory subunit gamma-1-like	0.5
K3YT86	calcium sensing receptor, chloroplastic-like	0.6
K3XDU6	ATP-dependent RNA helicase DHX36-like	0.5
K3YDC9	pentatricopeptide repeat-containing (PPR) protein	0.5
K3ZSE8	pentatricopeptide repeat-containing (PPR) protein	0.6
K3YUP4	aquaporin PIP2-4-like	0.5
K3Z2C7	peroxidase (POD) 1-like	0.5
K3Y890	peroxidase (POD) 1-like	0.2
K3Z7J4	cationic peroxidase SPC4-like	0.5
B Photosynthesis		
K3ZW79	chlorophyll a-b binding protein (LHCII), 7, chloroplastic-like	4.3
K3XL06	chlorophyll a-b binding protein (LHCII), 2, chloroplastic-like	3.5
K3ZJH2	chlorophyll a-b binding protein (LHCII) CP26, chloroplastic-like	1.5
K3XYV6	oxygen-evolving enhancer protein (OEE) 2, chloroplastic-like	1.5
K3Y8K2	quinone-oxidoreductase homolog, chloroplastic-like	2.3
K3YI45	quinone oxidoreductase-like protein, chloroplastic-like	1.9
X4ZEC4	Cytochrome b6	2.0
K4AFA9	photosystem I reaction center subunit II, chloroplastic-like	1.6
K4A024	ATP synthase subunit gamma, chloroplastic	1.7
K3ZA91	Ribulose bisphosphate carboxylase small chain	5.3
K3ZA66	Ribulose bisphosphate carboxylase small chain	4.6
B0YID3	Ribulose-1,5-bisphosphate carboxylase/oxygenase large subunit (Fragment)	1.5
K3Y7S0	Glyceraldehyde-3-phosphate dehydrogenase (GAPDH)	1.7
K3Y7X1	fructose-bisphosphate aldolase (FBA), chloroplastic-like	1.6
K3Z3Q6	pyruvate, phosphate dikinase (PPDK) 2	3.8
K3ZQE6	phosphoenolpyruvate carboxylase (PEPC) 2-like	1.7
K3XFH6	NADP-dependent Malic enzyme (ME)	2.9
K3XFV5	NADP-dependent Malic enzyme (ME)	1.7
K3YIQ9	carbonic anhydrase	1.8
K3XKA7	NADH-cytochrome b5 reductase-like protein	2.2
K3YZ82	ferredoxin-thioredoxin reductase	2.0
K3YS88	Thioredoxin reductase	1.5
K4A8E1	pheophorbide a oxygenase, chloroplastic-like	1.6
K3ZVU4	THYLAKOID FORMATION1, chloroplastic-like	1.5
K3Y6H7	15-cis-phytoene desaturase, chloroplastic/chromoplastic -like	1.6
K4ALQ8	protochlorophyllide reductase	1.5
K3YV15	chlorophyll a-b binding protein (LHCII) P4, chloroplastic-like	0.5
K4AEM5	photosystem I reaction center subunit III, chloroplastic-like	0.6
K3ZXZ6	photosystem I reaction center subunit psaK, chloroplastic-like	0.5
K3YI05	phosphoenolpyruvate/phosphate translocator 2, chloroplastic-like	0.6
K3ZXS7	thylakoid membrane phosphoprotein 14 kDa, chloroplastic-like	0.6
C Carbon metabolism		
K3XK17	fructokinase-1-like	1.6
K3YH95	glyceraldehyde-3-phosphate dehydrogenase (GAPDH)	2.4
K3YS38	glyceraldehyde-3-phosphate dehydrogenase (GAPDH)	1.6
K3ZIS7	fructose-bisphosphate aldolase (FBA)	1.6
K3XT03	fructose-bisphosphate aldolase (FBA) cytoplasmic isozyme-like	1.5
K3XXC5	Phosphoglycerate kinase	2.4
K3Z681	enolase 2-like	1.8
K4A8I4	multiple inositol polyphosphate phosphatase 1	2.2
K3Y755	pyruvate dehydrogenase E2 subunit	2.0
K3Z6H7	ATP-citrate synthase alpha chain protein 3	1.5
K3XFR6	ATP-citrate synthase beta chain protein 1	1.6
K4A754	lysosomal beta glucosidase-like	2.7
K3ZSG5	Glucose-1-phosphate adenylyltransferase	2.2
K4A2Q0	Beta-amylase	2.1
K3YQX5	pyrophosphate--fructose 6-phosphate 1-phosphotransferase subunit alpha-like	1.5
D ATP synthesis		
K3YVF1	ATP synthase subunit O, mitochondrial-like	2.1
K3Y764	apyrase 3-like	2.0
K3YT79	ADP,ATP carrier protein 2, mitochondrial-like	1.7
K4AIL8	Nucleoside diphosphate kinase (NDPK)	1.5
K3ZXB9	cytochrome c oxidase subunit 6b-1-like	2.1
K3ZED2	cytochrome c oxidase subunit 5b-1, mitochondrial-like	2.0
K3ZXZ1	Cytochrome b-c1 complex subunit 7	1.9
E Protein biosynthesis, folding and degradation		
K4AIU7	40S ribosomal protein S17-4-like	2.6
K3YLC1	60S acidic ribosomal protein P1	6.6
K3ZVC4	60S acidic ribosomal protein P0-like	2.6
K3YIN8	60S acidic ribosomal protein P0-like	2.5
K3YJD1	60S ribosomal protein L7-4-like	2.5
K3XZB6	60S ribosomal protein L35Ae	2.5
K3ZTV9	60S ribosomal protein L4-1-like	2.3
K3XME9	60S ribosomal protein L11-like	2.3
K3Y9P1	60S ribosomal protein L7-3-like	2.3
K4AI65	40S ribosomal protein S28-like	2.3
K3XLQ2	30S ribosomal protein S20	2.0
K4AAQ4	60S ribosomal protein L4-1-like	2.0
K3YWK9	40S ribosomal protein S24	1.9
K3YZ28	60S ribosomal protein L27a-2-like	1.8
K4AEV9	50S ribosomal protein L10, chloroplastic-like	1.8
K3XLM1	50S ribosomal protein L13, chloroplastic-like	1.7
K4AFY7	50S ribosomal protein L18, chloroplastic-like	1.7
K3XZH3	60S ribosomal protein L9-like	1.6
K3YJL9	Ribosomal protein	1.5
K3ZAW7	60S acidic ribosomal protein P2B-like	1.5
K3ZW60	40S ribosomal protein S3a	1.5
K3XM55	40S ribosomal protein S5-like	1.5
K3YN67	large subunit ribosomal protein L38	1.5
K3ZWN9	elongation factor 1-delta 1-like	1.9
K3ZWQ4	elongation factor 1-beta-like	1.6
K4ABL0	transcription elongation factor A protein 3-like	1.5
K3YGI3	glycine--tRNA ligase 1, mitochondrial-like	1.7
K3Y622	70 kDa peptidyl-prolyl isomerase-like	4.6
K3YU74	peptidyl-prolyl cis-trans isomerase	2.2
K3YYJ7	Peptidyl-prolyl cis-trans isomerase	1.7
K3YVL4	Peptidyl-prolyl cis-trans isomerase	1.6
K3XJ75	disulfide isomerase-like 2-2-like	1.5
K3XLF8	16.9 kDa class Iheat shock protein 1-like	4.7
K3YW49	18.6 kDa class III heat shock protein-like	4.7
K4AED3	26.7 kDa heat shock protein, chloroplastic-like	4.6
K3XQ86	17.5 kDa class II heat shock protein-like	4.2
K3XT07	17.5 kDa class II heat shock protein-like	3.7
K3Y9Z4	23.2 kDa heat shock protein-like	3.3
K3Y5K9	heat shock protein 82-like	3.2
K3ZJL1	21.9 kDa heat shock protein-like	3.1
K3XMX2	16.6 kDa heat shock protein-like	2.6
K3XMV8	16.9 kDa class I heat shock protein 1-like	2.0
K4A6U5	heat shock cognate 70 kDa protein-like	3.6
K4A6V7	heat shock cognate 70 kDa protein 2-like	1.8
K3Z4G5	heat shock 70kDa protein	1.8
K3XFF0	heat shock 70kDa protein	2.0
K3XWJ9	ASPARTIC PROTEASE IN GUARD CELL 2-like	1.9
K3Z2Q9	ATP-dependent Clp protease proteolytic subunit	1.8
K3ZHP9	Xaa-Pro aminopeptidase P-like	1.9
K3ZI70	Carboxypeptidase	1.8
K3Z5Z6	ASPARTIC PROTEASE IN GUARD CELL 2-like	0.6
K3Y7X0	cysteine proteinase 1-like	0.5
K3ZUJ8	cysteine proteinase 2-like	0.6
K3ZA06	60S acidic ribosomal protein L18a-like	0.6
F Metabolism-related proteins		
K3YQW0	Arginine decarboxylase	1.6
K3ZV28	spermidine synthase 1-like	1.5
K4A8M6	polyamine oxidase-like isoform X2	1.5
K3Y7I4	shikimate O-hydroxycinnamoyltransferase-like	1.5
K3YKY0	caffeic acid 3-O-methyltransferase-like	1.5
K3YI92	cinnamoyl-CoA reductase 1-like	1.5
K3YI97	O-methyltransferase ZRP4-like	2.2
K3ZIX3	O-methyltransferase 2-like	1.9
K3XEN1	delta-1-pyrroline-5-carboxylate synthase (P5CS)	1.5
K3YH74	betaine aldehyde dehydrogenase (BADH)1, chloroplastic-like	1.5
K3YRJ0	succinate-semialdehyde dehydrogenase, mitochondrial-like	2.9
K3Z7Y4	Cysteine synthase	1.7
K3YRQ5	3-isopropylmalate dehydratase-like	1.8
K3Z414	5-methyltetrahydropteroyltriglutamate--homocysteine methyltransferase-like	1.6
K4ADX7	peptide methionine sulfoxide reductase A4, chloroplastic-like	1.5
K3XGJ1	Serine hydroxymethyltransferase	1.5
K3XX32	Aspartate aminotransferase	1.5
K4A868	alanine aminotransferase 2-like	1.7
K4A1C4	alanine aminotransferase 2	1.5
K3XK90	chorismate mutase 3	1.6
K4AC07	omega-amidase NIT2-A-like	1.9
K3YRS9	allene oxide synthase (AOS), chloroplastic-like	1.7
K3ZV70	1-aminocyclopropane-1-carboxylate oxidase (ACO) 1-like	1.6
K3XV98	Lipoxygenase	2.8
K3ZI80	patatin	2.4
K4A844	3-ketoacyl-CoA synthase	2.0
K3ZQL2	cyclopropane fatty acid synthase	2.3
K3ZQQ2	acyl-CoA dehydrogenase family member 10-like	2.3
K3XGR5	O-acyltransferase WSD1-like	1.7
K3YK68	Acyl carrier protein	1.9
K3Z5V8	purple acid phosphatase (PAP) 2-like	2.7
K3XZ04	(DL)-glycerol-3-phosphatase (GPP) 2-like	2.2
K4A648	callose synthase 10-like isoform X2	1.5
K3ZN95	UDP-glycosyltransferase 72B3-like	1.6
K3XWQ1	UDP-glycosyltransferase 90A1-like	1.5
K3Z7G1	lactoylglutathione lyase	1.9
K3Y7S6	acetyl-CoA acetyltransferase	1.6
K3YIW6	lactoylglutathione lyase-like isoform X1	1.8
K3YGX1	4-coumarate--CoA ligase 1-like	0.5
K3Z9M3	methionine sulfoxide reductase B3, chloroplastic-like	0.6
K3YQA0	primary amine oxidase-like	0.6
K4A6W5	Asparagine synthetase	0.6
K3ZSH2	putative amidase C869.01-like	0.6
K3XH80	UDP-glycosyltransferase 88A1-like	0.6
K4A3F8	UDP-glycosyltransferase 90A1-like	0.6
G Cell organization-related proteins		
K4A9U8	tubulin alpha-1 chain-like	2.8
K3XWX5	tubulin beta-1 chain-like	2.8
H Others		
K3XME6	ADP-ribosylation factor 2-like	3.4
K3XHN1	WD-40 repeat-containing protein MSI4-like	1.6
K4ADU6	expansin-B3-like	1.7
K3ZVC8	plastid-lipid-associated protein 2	2.4
K4AB63	plastid-lipid-associated protein 3, chloroplastic-like	0.6

Protein accession: database accession numbers according to UniProt. Protein name: the function of differentially expressed proteins was annotated using the UniProt and NCBI database. T/C Ratio: relative fold change abundance of proteins in Foxtail millet under drought stress compared with control. (With a threshold of fold change (cutoff of over 1.5 for increased expression and less than 1/1.5 (0.67) for decreased expression) and T test p-value <0.01)

### Proteins function in stress and defense response

Under drought stress, the abundances of multiple stress and defense response proteins changed. These proteins mainly comprise late embryogenesis abundant (LEA) protein, ABA-, stress-and ripening-induced (ASR) proteins, plant nonspecific lipid transfer proteins (nsLTPs), 14-3-3 proteins, plant protease inhibitors, protein phosphatases, antioxidant enzymes and aquaporin (Table 1A).

As well-known drought response proteins, the abundances of four LEA proteins and two nsLTPs (K3YAY4, K3ZKC1) showed dramatic increases. The protein abundance of 13 ROS scavenging enzymes were also found to be increased under drought (Table 1A). Another 13 proteins showed decreased abundance (Table 1A), including receptor-like protein kinase, pentatricopeptide repeat-containing (PPR) protein and aquaporin.

### Photosynthesis, carbon metabolisms and ATP synthesis related proteins

**Fig. 5:**
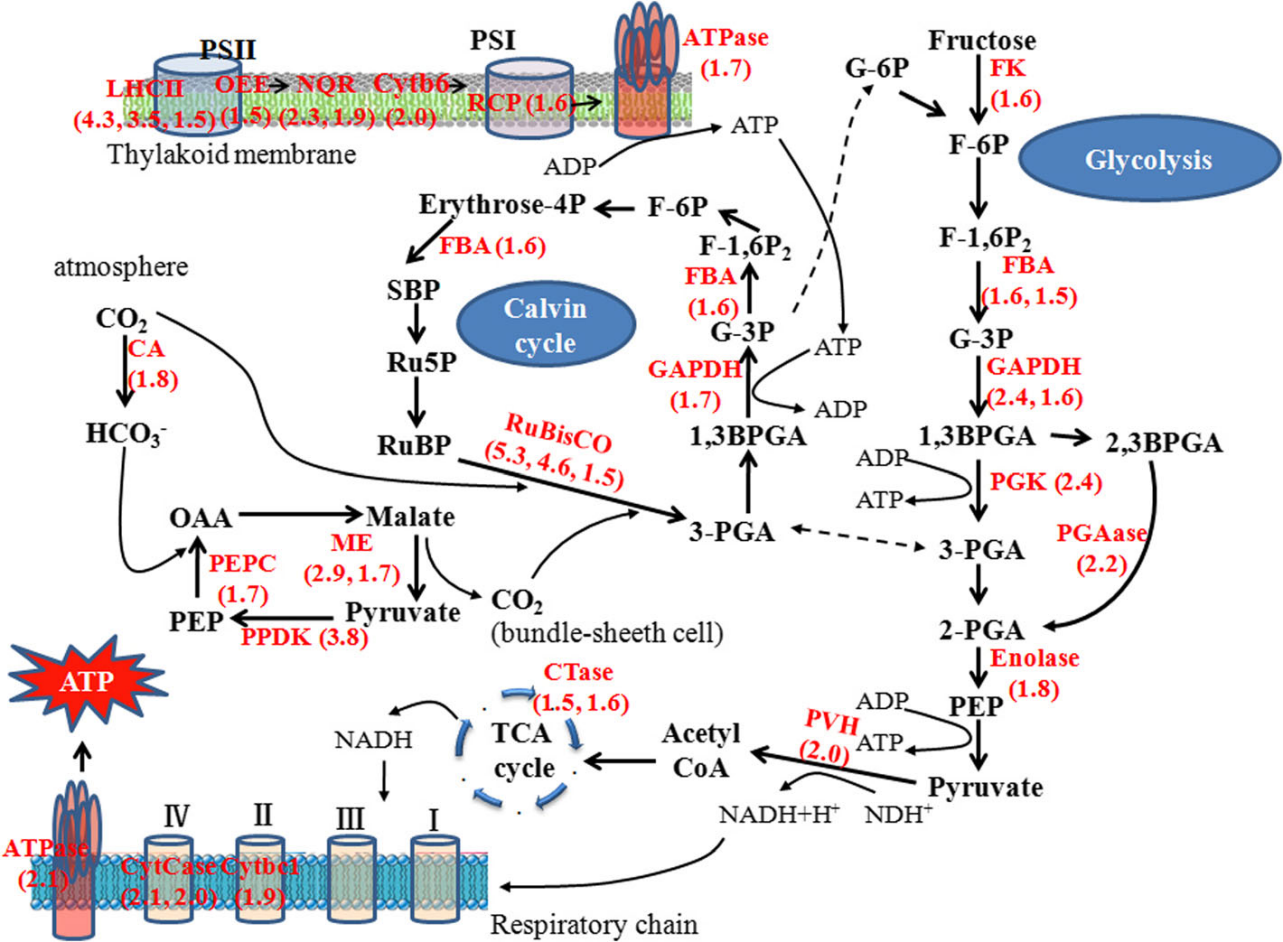
Schematic diagram of the identified the differential proteins participated energy metabolic pathways in foxtail millet seedlings under drought stress. All red words represent proteins with increased abundance and the change fold was also presented. The broken arrows indicate that there are multisteps between the two compounds. Abbreviations: F-6P: fructose-6-phosphate, F-1,6P_2_: fructose-1,6-bisphosphate, FBA: fructose-bisphosphate aldolase, G-3P: glyceraldehyde-3-phosphate, 1,3BPGA: 1,3-bisphosphoglycerate, PGK: phosphoglycerate kinase, GAPDH: glyceraldehyde 3-phosphate dehydrogenase, 2-PGA: 2-phosphoglycerate, 3-PGA: 3-phosphoglycerate, PGAase: 2,3-bisphosphoglycerate 3-phosphatase, FK: fructokinase, RuBisCO: ribulose-1,5-bisphosphate carboxylase/oxygenase, RuBP: ribulose-1,5-bisphosphate, CA: carbonic anhydrase, PVH: Pyruvate dehydrogenase, PEP: phosphoenolpyruvate, PEPC: phosphoenolpyrovate carboxylase, NADP^+^/NADPH: nicotinamide adenine dinucleotide phosphate, ME: NADP-dependent malic enzyme, SBP: Sedoheptulose-1,7-bisphosphate, S7P: Sedoheptulose-7P, R5P: Ribose-5P, Ru5P: Ribulose-5P, G-6p: Glucose-6P, ADP: adenosine diphosphate, ATP: adenosine triphosphate, PSI: photosystem I, PSII: photosystem II, RCP: reaction center protein, LHCII: chlorophyll a/b binding protein, LHC: light-harvesting complex, NQR: quinone-oxidoreductase, Cytb6: Cytochrome b6, CTase: citrate synthase, CytCase: cytochrome c oxidase subunit, Cytbc1: cytochrome b-c1 complex subunit, ATPase: ATP synthase

Self-protection processes, such as ROS scavenging, ion transport and osmolyte synthesis, have high ATP demands, and thus, several proteins involved in energy metabolism pathways are up-regulated under various abiotic stresses [30]. In our results, 50 such proteins related to photosynthesis, glycolysis, tricarboxylic acid (TCA) cycle and ATP synthesis for energy production were detected with obvious variation in abundance (Table 1B, C and D) (Fig. 5).

The abundance of 14 proteins that are involved in light reaction and Calvin Cycle were dramatically increased by more than 1.5 folds, such as three chlorophyll a/b-binding proteins (LHCII), one oxygen-evolving enhancer protein (OEE), two quinine oxidoreductases, two Rubisco small chain, and so on (Table 1B) (Fig. 5). The abundances of proteins involved in CO_2_ assimilation also increased, including pyruvate dehydrogenase, phosphate dikinase (PPDK), phosphoenolpyruvate carboxylase (PEPC), NADP-dependent malic enzyme (NADP-ME), and carbonic anhydrase (Table 1B) (Fig. 5).

In addition to photosynthesis-related proteins, the abundances of proteins related to carbohydrate and ATP metabolism also changed. These include 11 proteins involved in glycolysis and the TCA cycle, such as two glyceraldehyde-3-phosphate dehydrogenases (GAPDH), two fructose-bisphosphate aldolases (FBA), and two citrate synthases (Table 1C) (Fig. 5). Four proteins related to ATP metabolism, including ATP synthase subunit, apyrase, ADP/ATP carrier protein and nucleoside diphosphate kinase (NDPK), were all increased by over 1.5 fold (Table 1C) (Fig. 5).

### Proteins involved in protein biosynthesis, folding and degradation

**Fig. 6:**
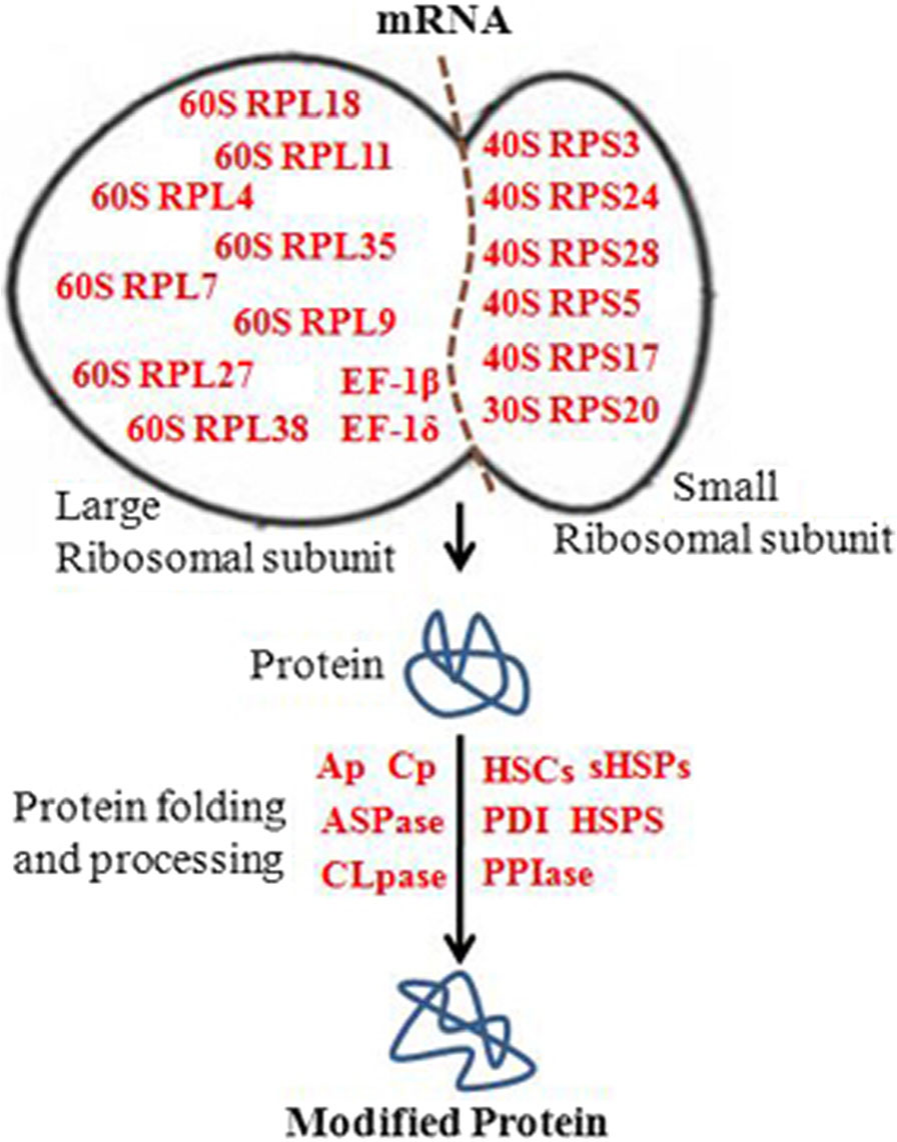
Schematic diagram of the identified the differential proteins participated protein biosynthesis, folding and degradation pathway in foxtail millet seedlings under drought stress. All red words represent proteins with increased abundance. Abbreviations: PDI: protein disulfide isomerase like protein, 40SRP: 40S ribosomal protein S6, HSC: heat shock cognate, HSP: heat shock protein, PPIase: Peptidyl-prolyl cis/trans isomerase, sHSPs: small HSPs, PDI: protein disulfide isomerase, ASPase: aspartic protease, CLpase: Clp protease proteolytic subunit, Ap: aminopeptidase, Cp: carboxypeptidase

The protein synthesis machinery performs a pivotal role in stress adaptation due to its role in post-transcriptional regulation. In our research, 52 proteins with marked variation related to protein biosynthesis, folding and degradation were identified (Fig. 6). From our proteomic data, the abundance of 21 ribosomal proteins with different sizes were identified to be significantly increased under drought stress (Table 1E). Peptidyl-prolyl cis-trans isomerase (PPIase) and disulfide isomerase are involved in protein folding and modification, respectively, and were found increased in their abundance, especially one PPIase, which increased by 4.6 fold (Table 1E). Heat shock proteins (HSPs) function as molecular chaperones; six HSPs and two HSC70 proteins showed significant accumulation in foxtail millet under drought stress (Table 1E). In addition, aspartic protease, Clp protease proteolytic subunit, aminopeptidase, and carboxypeptidase, which are responsible for removing modified, abnormal, and mistargeted proteins by proteolysis, were also up-regulated under drought stress (Table 1E) (Fig. 6).

### Metabolism-related proteins

In this research, the detected differential proteins were found to occupy a wide variety of metabolic pathways including polyamine metabolism, lignin biosynthesis, amino acid metabolism, secondary metabolism, hormone metabolism, and lipid and fatty acid metabolism (Table 1F). Here, three key enzymes involved in PA metabolism, such as arginine decarboxylase, spermidine synthase, and polyamine oxidase, and three proteins related to lignin biosynthesis, such as shikimate O-hydroxycinnamoyl transferase, caffeic acid 3-O-methyltransferase and cinnamoyl-CoA reductase, were all found to be increased in abundance after drought treatment (Table 1F). In addition, the proteins related to phosphorus metabolism, such as purple acid phosphatase (PAP), (DL)-glycerol-3-phosphatase (GPP), and callose synthase, also accumulated in foxtail millet under drought stress (Table 1F).

### Cell organization-related proteins

Microtubules (MTs) are one of the two key components in the eukaryotic cytoskeleton. The arrangements and stabilities of MTs are related to the stress resistance and tolerance of plants [31]. In foxtail millet, two proteins identified as tubulin α-1 chain and tubulin β-1 chain were both up-regulated by 2.8 folds (Table 1G). The increased abundance of α/β-tubulin in foxtail millet may affect polymerization and alignment of MTs, and further affect cell stability and plant resistance in response to drought.

### Other proteins changed under drought

In our proteomic analysis, other proteins, which have been reported to be involved in abiotic stress response, were also identified (Table 1H). The ADP-ribosylation factor (ARF), which plays critical roles in membrane trafficking, was increased by 3.4 folds in abundance when plants were stressed. The abundance of WD40 proteins and Expansins (EXP), which act as scaffolding molecules and mediate cell-wall loosening and extension, respectively, were increased by 1.6 folds in abundance (Table 1H). Plastid lipid-associated proteins (PAP) are amphipathic proteins and regulated by various abiotic stressors. Two PAPs with opposite accumulation trends were also identified (Table 1H).

### Variation of physiological parameters

**Fig. 7:**
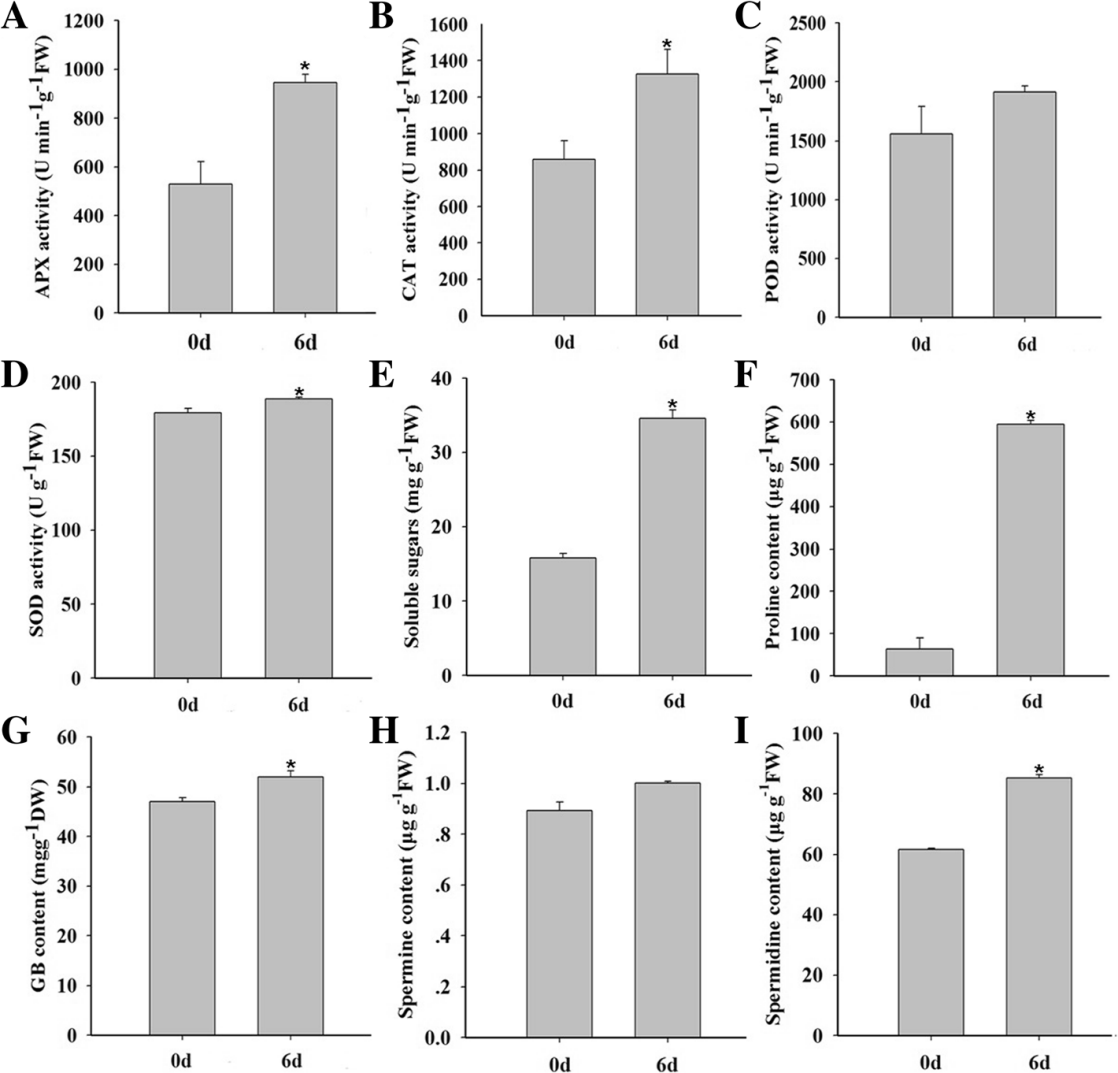
Physiologic parameters measurement in foxtail millet under drought stress. Total activity of the antioxidant enzymes APX (**a**), CAT (**b**), POD (**c**) and SOD (**d**) in foxtail millet under drought stress. The content of soluble sugare (**e**), proline (**f**), GB (**g**), spermine (**h**) and spermidine (**i**) in foxtail millet under drought stress

To further elucidate the molecular mechanism of drought tolerance in foxtail millet, physiological parameters, including antioxidant enzymes, the content of soluble sugar, proline, GB and polyamines, were measured. As shown in Fig. 7, after drought treatment, the activities of L-ascorbate peroxidase (APX), peroxidase (POD) and catalase (CAT) were increased 78%, 54% and 22%, respectively, compared with those of the control. There was only a slight increase in superoxide dismutase (SOD) activity. The enhanced activities of these antioxidant enzymes were consistent with the accumulation of the corresponding proteins in our proteomics data (Table 1A). The contents of proline and soluble sugars were about 9 and 2 folds higher than those of the control under drought respectively, and the GB content was also up-regulated. The spermine and spermidine levels in foxtail millet seedlings also increased by 12% and 38% under drought treatment, respectively.

### Validation of differential proteins by western blot

**Fig. 8:**
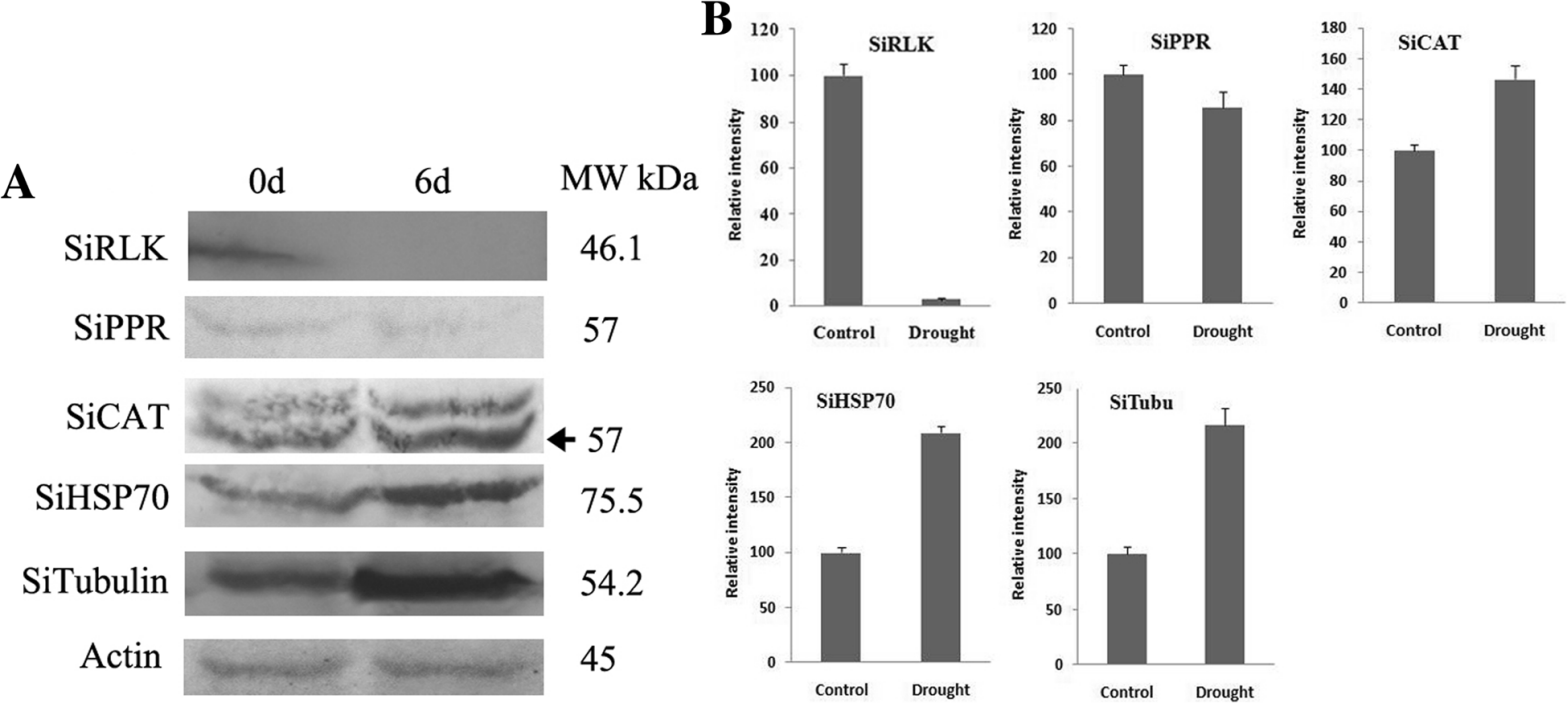
Western blot of selected drought-responsive proteins in foxtail millet. Five proteins including SiRLK(K3ZRC5), SiPPR (K3YDC9), SiCAT (K4A918), SiHSP70 (K4A6V7) and tubulin (K4A9U8) were chosen for western blot analysis. The SiActin was used as the internal control to confirm the same total protein loading quantity of different samples.  **a**: The result of western blot, **b**: Quantitative analysis of protein change using Image J

In order to validate the content variation of proteins identified in the proteomic analysis, five proteins including SiRLK (K3ZRC5), SiPPR (K3YDC9), SiCAT (K4A918), SiHSP70 (K4A6V7) and SiTubulin (K4A9U8) were randomly picked for western blot analysis. As shown in Fig. 8, drought stress could significantly induce the accumulation of SiCAT, SiHSP70 and SiTubulin, and obviously reduce the enrichment of SiRLK. The expression of SiPPR was slightly decreased in the drought treatment. The variation trends of these proteins under drought stress were in good accordance with the proteomic analysis results.

### Transcriptional analysis of drought-responsive proteins

**Fig. 9:**
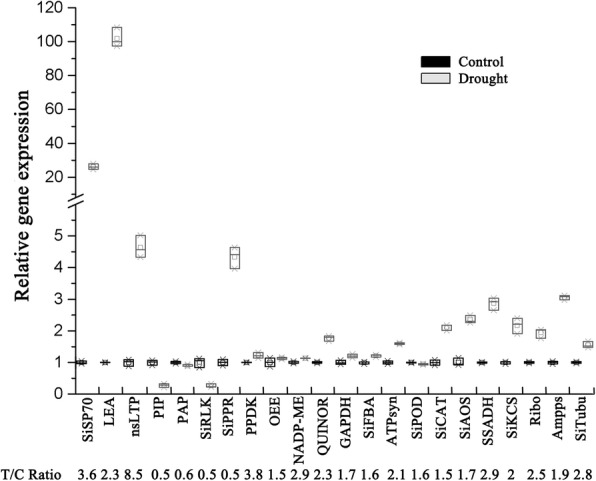
qRT-PCR analysis of gene expression patterns in Foxtail millet under drought stress. CK: control treatment. The SiActin gene was used as the internal control. The relative fold change abundance of corresponding proteins identified under drought stress compared with control treatment was presented in bottom line. NADP-ME: NADP-dependent malic enzyme, SiCAT: Catalase, SiLEA: late embryogenesis abundant protein, SiOEE: oxygen-evolving enhancer protein, GAPDH: glyceraldehyde-3-phosphate dehydrogenase, SiFBA: fructose-bisphosphate aldolase, QUINOR: quinone-oxidoreductase homolog, HSP70: heat shock protein, nsLTP: non-specific lipid-transfer protein, PPR: pentatricopeptide repeat-containing protein, Ribo: 60S ribosomal protein, SSADH: succinate-semialdehyde dehydrogenase, AOS: allene oxide synthase, PIP: aquaporin, ATPsyn: ATP synthase subunit O, PPDK: pyruvate, phosphate dikinase, Ampps: Xaa-Pro aminopeptidase, Tubu: tubulin, KCS: 3-ketoacyl-CoA synthase, PAP: plastid-lipid-associated protein, SiRLK: receptor-like serine/threonine-protein kinase

To explore the correlation between translation and transcription of the differential proteins and their coding genes during drought stress in foxtail millet, 22 differential proteins were randomly selected and qRT-PCR was conducted (Fig. 9). Of these 22 proteins, only eight proteins (PIP (K3YUP4), PAP (K4AB63), SiRLK (K3ZRC5), SiCAT (K4A918), SiAOS (K3YRS9), SSADH (K3YRJ0), SiKCS (K4A844), and Ribo (K3YJD1)) were positively correlated with their corresponding coding genes. Other proteins showed loosely correlated post-transcriptional and post-translational levels. As shown in Fig. 9, mRNA levels of *HSP70* and *LEA* were significantly increased by more than 25-fold after drought treatment, but their corresponding proteins were only increased by 3.3 and 2.3-fold, respectively. The post-transcriptional level nsLTP was up-regulated by 4.6 folds, but the abundance of its protein was increased by 8.5 folds. Other genes, such as *PPDK* (K3Z3Q6), *OEE* (K3XYV6), *NADP-ME* (K3XFH6), *QUINOR* (K3Y8K2), *GAPDH* (K3Y7S0), *SiFBA* (K3Y7X1), *ATPsyn* (K3YVF1), *SiPOD* (K3XJR1) and *SiTubu* (K4A9U8), showed no significant changes at the transcriptional level, even though they displayed differential expression profiles at the post-translational level. Furthermore, the expression pattern of *PPR* even exhibited an opposite trend compared to its coding protein under drought stress.

## Discussion

Drought stress is one of major constraints limiting crop production worldwide. This multidimensional stress causes changes in the physiological, morphological, biochemical, and molecular traits in plants. Many plants have evolved intricate resistance mechanisms to tolerate drought stress. However, these mechanisms are varied from plant species to species. Foxtail millet, as an extremely drought tolerant and resistant grain crop, has been proposed as a novel model species for functional genomic study and drought tolerance investigation [15, 32].

More and more research in foxtail millet subjected to drought stress demonstrate that functional proteins play key roles in stress response [33, 34]. Previous transcriptomic study suggested that an elaborate regulatory network positively regulated foxtail millet in response to drought. This network involved multiple biological processes and pathways, which included signal transduction, transcriptional regulation, redox regulation, photosynthesis, and osmotic adjustment [35]. However, The alterations in protein expression, along with the physiological variations occurring in foxtail millet under drought stress still require extensive and intensive research. In our study, 321 proteins that respond to drought were identified, and functional analysis revealed that these proteins were involved in stress response, photosynthetic and metabolic pathways (Table 1), indicating that multiple sophisticated mechanisms worked together to reestablish a new cellular homeostasis under drought stress.

LEA proteins are highly hydrophilic and thermally stable, the accumulation of LEA proteins may protect cellular structures from injuries by maintaining orderly structures within the cell [36]. Aquaporins are conserved integral membrane proteins that are involved in plant water uptake [37]. The decrease of aquaporin K3YUP4 under drought may be beneficial to reduce membrane water permeability, and promote cellular water conservation.

The antioxidant defense system in plants under drought stress is composed of ROS scavenging enzymes. Among them, CAT, SOD, APX, and GPx are essential to remove ROS and act synergistically to counteract oxidative damage caused by drought stress [38]. In our results, the abundance of 13 ROS scavenging enzymes and activities of APX, POD, CAT and SOD increased (Table 1A and Fig. 7), all of which could enhance drought resistance in foxtail millet

The abundance of ribosomal proteins, protein chaperones, and proteases were all changed significantly, all of which perform pivotal roles in protein synthesis and modification, aiding in stress adaptation. From our data, 21 ribosomal proteins were identified to be significantly increased under drought (Table 1E, Fig. 5). Similar results have also been reported in cucumber and maize under stres [39]. HSPs and HSC70 were identified to be significant accumulated which could aid in maintaining protein homeostasis and promoting refolding of denatured proteins under drought (Table 1E) [40, 41]. The four proteases, which could participate in proteins modification and correstions, were also be identified accumulated under drought (Table 1E). These differential proteins and response mechanisms are consistent with previous studies in *Arabidopsis* under salt stress [42].

The maintenance of photosynthetic rates under drought stress is essential for drought tolerance in crops [43]. Here the increase in multiple light reaction-related proteins and Calvin Cycle-related proteins (K3ZA91 and K3ZA66) were identified (Fig. 5) [44]. Previously reported that drought stress could lead to a reduction in internal CO_2_ concentration due to stomatal closure [44]. The increase in the CO_2_ assimilation-related proteins may facilitate CO_2_ concentration and assimilation, and in turn contribute to maintain higher photosynthetic rate in foxtail millet under drought stress (Table 1B) (Fig. 5).

The glycolytic pathway has also been reported to respond to drought. In our results, the expression of fructokinase was up-regulated 1.6 fold, and the amounts of related enzymes were all increased, which indicates a potential increased flux of carbohydrates to the TCA cycle (Table 1C) (Fig. 5) [39, 45, 46]. This is further supported by the increase we saw in pyruvate dehydrogenase and citrate synthase; pyruvate dehydrogenase catalyzes the oxidative decarboxylation of pyruvate into acetyl-CoA and NADH and links the glycolysis pathway to the TCA cycle, and citrate synthase catalyzes the first step of the TCA cycle. The results indicate an acceleration of carbon metabolism, which would provide more energy for stress resistance [28]. The enhanced energy production resulted in decreaesd carbohydrate synthesis, which leaded to a reduction in biomass under drought stress.

The reversible protein phosphorylation regulated by kinase and phosphatase is fundamental for many signaling pathways of various stress-related biological processes [47]. Our study detected the variation of one serine/threonine-protein phosphatase 2A and four receptor-like protein kinases (Table 1A). Serine/threonine protein phosphatase 2A is significant in its role as a regulator for microtubule-associated proteins (MAPs). This, taken along with the increase in α/β-tubulin, suggests that the phosphorylation and stabilizing microtubules are key factors in drought response of foxtail millet [48].

Abundance of proteins belonging to different metabolic pathways, including polyamine metabolism, lignin biosynthesis, fatty acid metabolism, amino acid metabolism, hormone metabolism and nucleotide metabolism (Table 1F), were also affected under drought [49]. In the abiotic stress response, polyamines, ROS (H_2_O_2_) and NO act synergistically in promoting ABA responses in guard cells [21, 50, 51]. In our study, three key enzymes involved in PA metabolism were found to be accumulated (Table 1F). The increase in PA synthetases were consistent with the accumulation of polyamines (Fig. 6).

Osmotic regulated substances, such as proline and GB, could improve water uptake from drying soil and protect cell metabolism by balancing the osmotic pressure and maintaining cell turgor in plant cells during drought [52, 53]. Delta-1-pyrroline 5-carboxylase synthetase (P5CS) (K3XEN1) and betaine aldehyde dehydrogenase (BADH) (K3YH74), which catalyze the rate-limiting steps of proline and glycinebetaine (GB) biosynthesis respectively, were both significantly up-regulated to deal with osmotic stress caused by drought (Table 1F).

Lignin is a basic component of the plant secondary cell wall, and the lignin content and composition changed during abiotic and biotic stresses [54]. Here, three proteins related to lignin biosynthesis such as shikimate O-hydroxycinnamoyl transferase, caffeic acid 3-O-methyltransferase and cinnamoyl-CoA reductase were found to be increased, while only one protein, 4-coumarate--CoA ligase, was reduced under drought (Table 1F). Very long chain fatty acids (VLCFAs) are important components of the protective barrier present at the plant-environment interface [55]. 3-ketoacyl-CoA synthase (KCS), which is responsible for VLCFA synthesis, was found to be up-regulated about two folds after drought treatment. A cyclopropane fatty acid synthase was also increased 2.3 folds (Table 1F). Previous evidence has suggested that the existence of a cyclopropane ring within membrane fatty acids could enhance the stability of biological membranes [56]. NsLTPs are soluble, small, basic proteins in plants, which have been reported to be involved in catalyzing the transfer of phospholipids and play an important role in plant defense and stress responses [57]. Our results suggest a very important role of lipid metabolism in drought tolerance of foxtail millet, which could also explain the dramatic increase of nsLTP (K3YAY4) under drought. Further in-depth study will help to understand how lipids function in the stress-resistance mechanisms in foxtail millet.

The plant hormones, especially jasmonic acid (JA) and ethylene (ETH), are important signaling molecules and play critical roles in mediating biotic/abiotic stress responses in plants [58]. In this research, the key enzymes of allene oxide synthase (AOS) (K3YRS9) and 1-aminocyclopropane-1-carboxylate oxidase (ACO) (K3ZV70), which catalyze the first and final step of JA and ETH biosynthesis respectively, were all increased in abundance under drought (Table 1F). These results further proved that the phytohormone metabolic pathway also participates in stress responsive processes.

We also performed correlated analysis of transcriptomics−proteomics, in which the corresponding transcripts of only 23 differential proteins were identified in our proteomics study (Additional file 7: Table S6) [15]. The weak correlation between the transcriptomic and proteomic data was consistent with the results of previous reports in other species. As protein isoforms and their abundances are not only regulated at the transcription level, but also at post-transcriptional, translational, and post-translational levels. The results confirmed the formerly established perception that transcription levels are not directly correlated with protein expression levels [14, 28, 39, 46], and post-transcriptional regulation performs a vital role in gene expression [59].

It could be concluded from this study that the proteins that participated in energy metabolism and protective materials synthesis were obviously changed, and the contents of most of them were enhanced dramatically. These changes further contribute to protect plant cells avoiding water defect damage, and maintain growth of foxtail millet under drought. These were consistent with previous proteomic studies of drought tolerant genotype plants under drought [43].

In all, the proteomic profiling obtained in this research has offered comprehensive insights into the various mechanisms of drought tolerance in foxtail millet, and more detailed studies are needed to determine the key proteins and pathways of plants under stress.

## Conclusions

**Fig. 10:**
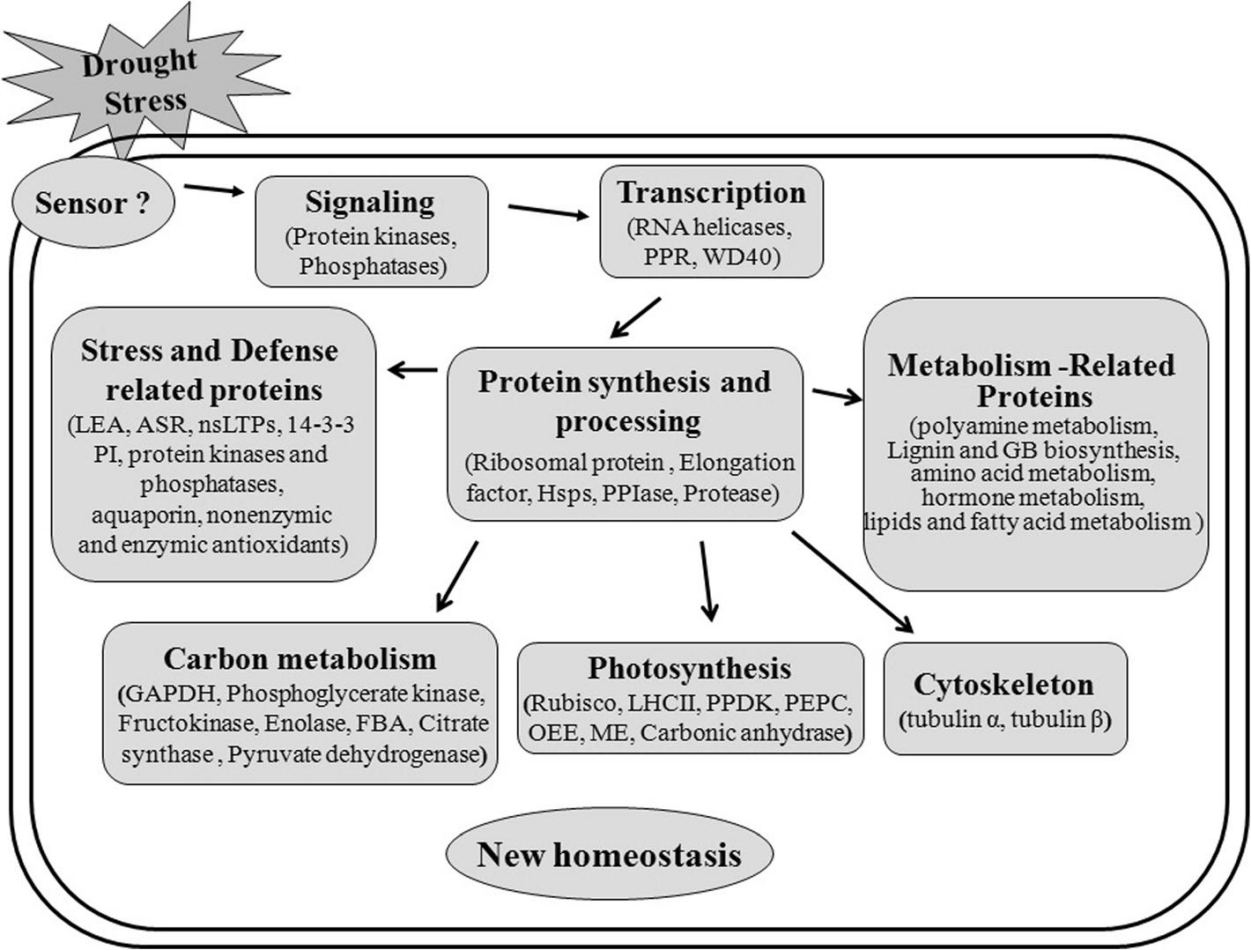
A simple model of the drought stress responses in foxtail millet. Foxtail millet can perceive duought stress signals by putative sensors and transmit them to the cellular machinery by signal transduction to regulate gene expression. Through regulation of transcription, protein synthesis and processing, the abundance and activities of functional proteins involved in stress and defense response, energy pathways and a variety of metabolism pathways were changed. These processes might work cooperatively to establish a new cellular homeostasis under drought stress

In this report, quantitative proteomic analysis was employed and a series of comprehensive proteomic data of foxtail millet under drought stress was obtained. Among the identified proteins, 252 up-regulated proteins showed more than a 1.5-fold change and 69 proteins showed less than 0.67-fold change in abundance. These proteins were further classified into a broad range of biological processes, particularly participation in stress and defense responses, photosynthesis, carbon metabolism, protein synthesis and various metabolic processes. Drought signals are perceived and transmitted into the cell, which is mediated by putative sensors and signal transduction mechanisms. Through post-transcriptional, translational, and post-translational regulation, the abundance and activities of functional proteins involved in stress and defense responses, energy pathways and a variety of metabolic pathways were changed to reestablish a new cellular homeostasis under drought stress (Fig. 10). Notably, most of the proteins identified in this work have previously been found to participate in responses to and be regulated by drought and other abiotic stresses. Western blot and qRT-PCR were performed to analyze the accumulation and expression of proteins and mRNA of the selected proteins, respectively. Stress related physiological parameters, such as antioxidant enzymes, the content of soluble sugar, proline, GB and polyamines, were also detected for foxtail millet after drought treatment.

The proteome profiling obtained in this research has offered comprehensive insights into the various mechanisms of drought tolerance in foxtail millet. The identified differential proteins are candidate resources for the millet research community in selecting proteins for further stress-tolerant germplasm innovation and cultivation.

## Additional files

Additional file 1: **Figure S1.** The RNA Electropherogram. 1μg of total RNA was separated by denaturing 1.0% (w/v) agarose gel, and stained with ethidium bromide. Total RNA was isolated from foxtail millet seedlings, 1: Control treatment, 2: drought treatment, M: DNA Maker. (JPG 21 kb)

Additional file 2: **Table S1.** Oligonucleotide primers used in qRT-PCR in this study. (DOC 98 kb)

Additional file 3: **Table S2.** List of annotation all 4075 identified proteins. (XLSX 1117 kb)

Additional file 4: **Table S3.** List of 2474 quantified proteins were found in all four replicates in foxtail millet after drought treatment. (XLS 97 kb)

Additional file 5: **Table S4.** List of 321 differentially expressed proteins in foxtail millet after drought treatment. (XLSX 23 kb)

Additional file 6: **Table S5.** The parameters of Protein-Protein Interactions (PPIs) Networks (XLSX 118 kb)

Additional file 7: **Table S6.** Comparative analysis the transcriptome and proteome data in foxtail millet after drought treatment. (XLSX 17 kb)

## Abbreviations

APX: Ascorbate peroxidase
CAT: Catalases
FA: Formic acid
FBA: Fructose-bisphosphate aldolase
GAPDH: Glyceraldehyde-3-phosphate dehydrogenase
GB: Glycine betaine
GO: Gene Ontology
GPP: (DL)-glycerol-3-phosphatase
GST: Glutathione S-transferase
HSP: Heat shock protein
KEGG: Kyoto Encyclopedia of Genes and Genomes
lncRNA: Long noncoding RNA
MT: Microtubule
NDPK: Nucleoside diphosphate kinase
OEE: Oxygen-evolving enhancer protein
PAP: Purple acid phosphatase
POD: Peroxidase
PPIase: Peptidyl-prolylcis/transisomerase
siRNA: Small interfering RNA
SOD: Superoxide dismutase
