# Supplementary figures and images for: Comparative proteomic investigation of drought responses in foxtail millet
Source: BMC Plant Biol. 2018 Nov 29;18:315. doi: 10.1186/s12870-018-1533-9 (PMC6267058; doi:10.1186/s12870-018-1533-9)

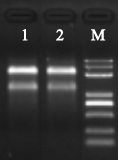

Supplement: Supplementary file 1 — Figure S1. The RNA Electropherogram. 1μg of total RNA was separated by denaturing 1.0% (w/v) agarose gel, and stained with ethidium bromide. Total RNA was isolated from foxtail millet seedlings, 1: Control treatment, 2: drought treatment, M: DNA Maker. (JPG 21 kb) [file 12870_2018_1533_MOESM1_ESM.jpg]
