# Supplementary material for: Comparative proteomic investigation of drought responses in foxtail millet
Source: BMC Plant Biol. 2018 Nov 29;18:315. doi: 10.1186/s12870-018-1533-9 (PMC6267058; doi:10.1186/s12870-018-1533-9)
Supplement: Supplementary file 2 — Table S1. Oligonucleotide primers used in qRT-PCR in this study. (DOC 98 kb) [file 12870_2018_1533_MOESM2_ESM.doc]

Table S1 List of the sequences of the primers used in qRT-PCR.

| Protein  accession | NCBI accession | Primer | Sequence (5'-3') | Annealing temperature | Amplicon size |
| --- | --- | --- | --- | --- | --- |
|  | XM_004978702 | SiActin1-S1 | CGCATATGTGGCTCTTGACT | 57℃ | 126bp |
|  |  | SiActin1-A1 | GGGCACCTAAATCTCTCTGC |  |  |
| K3XFH6 | XM_004968410 | NADP-ME-S1 | GGCTTCCTTCAATGAGAG | 57℃ | 91bp |
|  |  | NADP-ME-A1 | GGTCCAGTTATATGCTTGT |  |  |
| K3XJR1 | XM_004968838 | SiPOD-S1 | TTGGTTCAGAGACACTTG | 57℃ | 145bp |
|  |  | SiPOD-A1 | ATACATACACAGTTGAAGAGA |  |  |
| K3XLP0 | XM_004969628 | SiLEA-S2 | TACTAAGATGCTCGTGTG | 57℃ | 86bp |
|  |  | SiLEA-A2 | CTGAAATTACTTTGAACCTAAAC |  |  |
| K3XYV6 | XM_004965743 | SiOEE-S2 | GACAAGAGGTGGTTCAAG | 57℃ | 83bp |
|  |  | SiOEE-A2 | AAGGAAAAGAATGCTTATGC |  |  |
| K3Y7S0 | XM_004975790 | GAPDH-S1 | CCTCGCATTGTAATAACC | 57℃ | 103bp |
|  |  | GAPDH-A1 | GAATTGTTGGGAGTTTGG |  |  |
| K3Y7X1 | XM_004977197 | SiFBA-S2 | ACTCCATTCGTTGTTCAC | 57℃ | 82bp |
|  |  | SiFBA-A2 | ATGCTCTTGCTGTCTTCT |  |  |
| K3Y8K2 | XM_004975287 | QUINOR-S1 | TAACAGCAGCAAGAAGTA | 57℃ | 99bp |
|  |  | QUINOR-A1 | TATAAACATCTATCCTCAAACC |  |  |
| K3Y9Z4 | XM_004975681 | HSP23-S1 | AATTAGCAACCTCAATCG | 57℃ | 95bp |
|  |  | HSP23-A1 | CATCATCACTGGTCCAAG |  |  |
| K3YAY4 | XM_004977323 | nsLTP-S1 | CGTCGCTACATACCTATAC | 57℃ | 124bp |
|  |  | nsLTP-A1 | GACTCCTCTCTCACACAT |  |  |
| K3YDC9 | XM_004978236 | PPR-S1 | ATTATCTGCTTATGTCCAA | 57℃ | 130bp |
|  |  | PPR-A1 | GTTCCACCAATCTCTATC |  |  |
| K3YJD1 | XM_004972464 | Ribo-S1 | CGAACCTGAAGAGTGTTA | 57℃ | 82bp |
|  |  | Ribo-A1 | TTGTTGCTCAGAGGAATC |  |  |
| K3YRJ0 | XM_004951692 | SSADH-S1 | CGTAACAGTGGACAGACA | 57℃ | 95bp |
|  |  | SSADH-A1 | TGAACAGCCTTGATGAATG |  |  |
| K3YRS9 | XM_004951166 | AOS-S1 | TTCACCAAGCTCGTCAAG | 57℃ | 111bp |
|  |  | AOS-A1 | ATCGGAACACAATTACACAT |  |  |
| K3YUP4 | XM_004953115 | PIP-S1 | CCTCACACACCACTCTAG | 57℃ | 84bp |
|  |  | PIP-A1 | ATCAGTACCTTAGCTTGC |  |  |
| K3YVF1 | XM_004953588 | ATPsyn-S1 | GAAGGTGTTTGATATGTC | 57℃ | 112bp |
|  |  | ATPsyn-A1 | CACTGTATTCTGATGATG |  |  |
| K3Z3Q6 | XM_004962073 | PPDK-S1 | GAAGGAATTGGACTATGC | 57℃ | 80bp |
|  |  | PPDK-A1 | ATAATCATCTGCCTCACA |  |  |
| K3ZHP9 | XM_004979350 | Ampps-S2 | TTCAATCAGATGCCAGTC | 57℃ | 109bp |
|  |  | Ampps-A2 | GAGCCAGAATCAATCCAA |  |  |
| K4A9U8 | XM_004981865 | Tubu-S1 | CTTGTCTTCAATGCTGTT | 57℃ | 87bp |
|  |  | Tubu-A1 | CTTCTTGCCATAGTCAAC |  |  |
| K4A844 | XM_004960769 | KCS-S1 | GATGGTGGTCTCTGTCTA | 57℃ | 75bp |
|  |  | KCS-A1 | ATGGATCATCGGTAGCATA |  |  |
| K4AB63 | XM_004983497 | PAP-S1 | AGCATATTCTGAACTCCT | 57℃ | 113bp |
|  |  | PAP-A1 | TTGAAGCATTGACTATCG |  |  |
| K3ZRC5 | XM_004956247 | SiRLK35S2 | AATCTGTCAATCATCTGCGTAG | 57℃ | 82bp |
|  |  | SiRLK35A2 | CGTCTCTTCCAATCCTCAAC |  |  |
